# The relationship between triglyceride/high-density lipoprotein cholesterol ratio and coronary microvascular disease

**DOI:** 10.1186/s12872-023-03229-4

**Published:** 2023-05-02

**Authors:** Li ping Liao, Lei Wu, Yang Yang

**Affiliations:** 1 Cardiology Department, Jiading Branch of Shanghai General Hospital, Shanghai Jiaotong University School of Medicine, 800 Huangjiahuayuan Road, Shanghai, 201803 China; 2 Department of Cardiology, The First Affiliated Hospital of Anhui Medical University, Hefei, Anhui, 230000 China

**Keywords:** Coronary microvascular disease, Coronary angiography, Insulin resistance, Triglyceride/high-density lipoprotein cholesterol ratio

## Abstract

**Background:**

As a novel marker of insulin resistance, the ratio of triglyceride/high-density lipoprotein cholesterol (TG/HDL-C) has been recently reported to be related to the occurrence of coronary artery diseases. However, no research has been conducted to probe whether the TG/HDL-C ratio is associated with the occurrence of coronary microvascular disease (CMVD).

**Aim:**

This study investigates the association between the TG/HDL-C ratio and the occurrence of CMVD.

**Methods:**

This study included 175 patients diagnosed with CMVD in the Department of Cardiology of our hospital from October 2017 to October 2021 as the study group and 175 patients with no chest pain, no history of cardiovascular disease and drug use, and negative results of exercise treadmill testing as the non-CMVD group. The clinical data of the two groups were compared. In addition, the risk factors of CMVD were analyzed with logistic regression, and the efficacy of independent risk factors in predicting CMVD was analyzed with a receiver operating characteristic (ROC) curve.

**Results:**

Compared with those in the non-CMVD group, the proportion of females, the incidence of hypertension and type 2 diabetes, the level of platelet count, TG, and C-reactive protein, and the ratio of TG/HDL-C were increased in the CMVD group, accompanied by decreased levels of albumin and HDL-C (*P* < 0.05). Logistic regression results revealed C-reactive protein (the area under the ROC curve [AUC] value: 0.754; 95% confidence interval [CI]: 0.681–0.827), sex (the AUC value: 0.651; 95%CI: 0.571–0.730), albumin (the AUC value: 0.722; 95%CI: 0.649–0.794), and TG/HDL-C ratio (the AUC value: 0.789; 95%CI: 0.718–0.859) as the independent risk factors of CMVD.

**Conclusion:**

The TG/HDL-C ratio is an independent risk factor for the occurrence of CMVD.

## Introduction

The coronary artery system is composed of three important parts, including the large epicardial coronary artery, anterior arteriole, and arteriole. Specifically, the nearest epicardial coronary artery is about 0.5–5.0 mm in diameter and is the generalized coronary artery. The anterior arteriole is located in the middle part, whose diameter is approximately 100–500 μm. Arterioles of < 100 μm in diameter constitute the distal portion of the coronary artery, which is the last part. As reported, the stenosis of epicardial coronary arteries is associated with the occurrence of several diseases, such as angina pectoris and myocardial ischemia. In addition, mounting studies have confirmed that coronary microvascular disease (CMVD) can also lead to coronary artery blood flow disorder and myocardial ischemia [1].

CMVD refers to a clinical syndrome with objective evidence of angina pectoris or myocardial ischemia resulting from abnormal structure or function of anterior arterioles and arterioles. As reported, over 35% of patients with stable angina pectoris and around 10–15% of patients with acute coronary syndrome exhibit no large epicardial coronary artery occlusion following coronary angiography [2] and are ultimately diagnosed with CMVD. Moreover, CMVD is associated with an increased incidence of major adverse cardiovascular events, which seriously affects the prognosis of CMVD patients [3].

CMVD lacks specific clinical manifestations since it primarily manifests as angina pectoris, which underlies the difficulty in distinguishing CMVD from angina pectoris induced by obstructive coronary artery disease. Currently, exercise stress testing is the traditional method for the diagnosis of CMVD [4]. Nevertheless, the study by Mygind et al. showed the low sensitivity and specificity of exercise stress testing for the diagnosis of CMVD [5]. Additionally, cardiac magnetic resonance, positron emission tomography, transthoracic doppler echocardiography, and dynamic myocardial perfusion computed tomography can be used to evaluate coronary vascular function. Regrettably, the use of these examination methods in clinical practice is greatly limited by their high cost, complex operation, and high radiation dose. Therefore, it is of enormous significance to find a simple indicator that can predict CMVD occurrence in patients with angina pectoris or high-risk factors for coronary artery disease.

As risk factors for cardiovascular disease, dyslipidemia is attributable to the imbalance of triglycerides (TG), low-density lipoprotein cholesterol (LDL-C), and high-density lipoprotein cholesterol (HDL-C), which are critical constituents of human lipid fraction [6]. Of note, an epidemiological study revealed that lipid-related ratio complexes were superior to single lipid markers in predicting cardiovascular disease risk [7].

The TG/HDL-C ratio was first identified by Gaziano et al. [8] as an indicator of atherosclerosis. Furthermore, a subsequent study exhibited that the TG/HDL-C ratio had better potential than other blood lipid indicators in predicting insulin resistance and the levels of atherogenic small and low-density lipoproteins [9]. In addition, another study demonstrated that the TG/HDL-C ratio was more valuable than other single lipid markers because it could reflect the complex interaction between lipoprotein metabolism and better predict the occurrence of plasma atherosclerosis [1]. A growing body of evidence supports that the TG/HDL-C ratio, in addition to its simplicity and practicality, carries predictive value for cardiovascular events and may be of clinical implication.

At present, no studies on the association between the TG/HDL-C ratio and CMVD are available. In this study, we, therefore, delved into the association between the TG/HDL-C ratio and CMVD, thus providing novel ideas for the early diagnosis of CMVD in patients with angina pectoris and then improving the prognosis of CMVD patients.

## Methods

In this research, we selected 705 patients with suspected angina pectoris who underwent coronary angiography from October 2019 to October 2021 in our hospital.

The exclusion criteria of patients were as follows: patients previously undergoing percutaneous coronary intervention surgery and coronary artery bypass transplantation; patients with new myocardial infarction within 3 months and valvular heart disease (mitral stenosis, mitral regurgitation, aortic stenosis, and aortic incompetence); patients with cardiomyopathy (dilated cardiomyopathy, hypertrophic cardiomyopathy, restrictive cardiomyopathy, and myocarditis), pulmonary thromboembolism, anemia, active bleeding, or bleeding tendency ;lipid-lowering treatment or drugs that affect blood lipids before admission; patients with severe hepatic and renal insufficiency: alanine aminotransferase or aspartate aminotransferase ≥ 3 times the upper limit of normal and glomerular filtration rate < 60 mL·min^− 1^·(1.73 m^2^)^−1^; patients with severe heart failure (left ventricular ejection fraction ≤ 35%), left ventricular hypertrophy (left ventricular wall ≥ 12 mm in echocardiography); patients with blood system diseases, malignant tumors, severe infections, trauma, and autoimmune diseases.

All patients discontinued vasoactive drugs (calcium antagonists, angiotensin-converting enzyme inhibitors, angiotensin receptor blockers, and nitrates) at least 5 days before study enrollment.

Additionally, 388 healthy subjects were selected from the population with negative results of exercise treadmill testing, no chest pain, and no history of cardiovascular disease and drug use in the physical examination center of our hospital in 2021. The age and gender of control subjects were adjusted with the propensity score matching method, and, eventually, 175 subjects were selected as the control group.

All procedures were carried out in accordance with the *Declaration of Helsinki* and under approval from the human ethics committee of each institution. After being fully informed of the purpose of the research, all participants signed a written consent form to agree to the use of their medical information for the research.

### Definition of CMVD

**Fig. 1:**
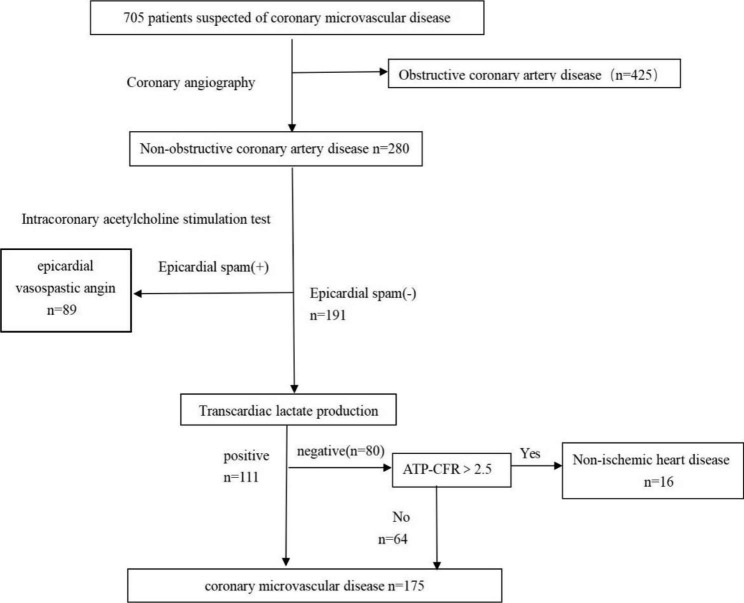
The diagnosis of coronary microvascular disease

705 patients suspected of coronary microvascular disease underwent coronary angiography. CMVD was defined as not only without epicardial spasm but also lactic acid levels between intracoronary and coronary sinuses in ACh-provocation test were increased (the results are positive) or adenosine triphosphate induced coronary flow reserve ratio (ATP-CFR) was below 2.5 if lactic acid levels were decreased.(The specific diagnosis process is described in Fig. 1.)

### Angiographical identification of epicardial spasm

The positive coronary angiography (CAG) result of coronary artery spasm in the ACh-provocation test was defined as “transient, complete, or subtotal occlusion (≥ 90% stenosis)”.

### Lactate measurement in the ACh-provocation test

Myocardial ischemia was assessed by detecting increased production of lactate in coronary artery circulation. Coronary sinus lactate production was measured with the ACh stimulation test only in left coronary arteries because the coronary sinus discharges blood from the left coronary artery system rather than from the right coronary artery system. Paired blood samples were simultaneously collected with coronary sinus catheters from aortic roots (ARs) and coronary sinuses (CSs) at three time points: when no drug was administered to left coronary arteries, when 100 µg ACh was administered to left coronary arteries, and when isosorbide dinitrate (ISDN) was administered to left coronary arteries.

Lactic acid yields were calculated with the following formula: [lactate (AR) – lactate (CS)]/lactate (AR) × 100(%). This ratio is negative in subjects without myocardial ischemia and positive in patients with myocardial ischemia.

### Intracoronary ACh-provocation test

ACh chloride (20 µg,50 µg, and 100 µg) was injected into the left coronary artery three times within 30 s, followed by CAG 1 min after each stimulation. Each dose of ACh chloride was administered at an interval of 5 min. Subsequently, 50 µg ACh was injected into the right coronary artery without the FloWire before CAG. After the administration of ISDN and coronary angiography at an interval of 10 min, ATP (150 µg/kg/min) was administered via the central vein until maximal hyperemia was achieved. Next, ATP-CFR was calculated with the following formula: average peak velocity (AVP; hyperemia)/APV (after ISDN administration).According to the guidelines of the Japanese Circulation Society, adenosine triphosphate–induced coronary flow reserve (ATP-CFR) scores also were measured for the diagnosis of microvascular coronary dysfunction in non-obstructive coronary atherosclerotic heart disease.

### Data collection

General clinical data were collected from the electronic medical record system after patients were admitted to the hospital. Elbow venous blood (4 mL) was obtained from each enrolled patient on an empty stomach on the day post-admission, followed by routine blood and biochemical index tests in anticoagulant tubes. Hypertension was diagnosed based on the standard definition of the 2020 international society of Hypertension Global Hypertension Practice Guidelines [10], while type 2 diabetes was diagnosed as per the recommendations of the American Diabetes Association [11].

### Statistical analysis

SPSS 26.0 data software was utilized for statistical analysis. Initially, the normal distribution of all measurement data was tested. Then, the data conforming to normal distribution were exhibited as mean ± standard deviation and compared between the two groups with the *T*-test. Data with skewed distribution were summarized as M (P25, P75) and compared between the two groups with the non-parametric test. Count data were displayed as example (%) and compared between the two groups with the rank sum test. The risk factors of CMVD in the enrolled patients were analyzed with multivariate logistic regression. A receiver operating characteristic (ROC) curve was plotted to evaluate the diagnostic efficiency of the TG/HDL-C ratio for CMVD. All tests were two-sided, and a difference with *P* < 0.05 was considered statistically significant.

## Results

### Comparison of clinical data between the two groups

There was no significant difference between the CMVD and non-CMVD groups in terms of age, body mass index, heart rate at admission, and the proportion of smokers, alcohol drinkers, and patients with left ventricular ejection fraction of 35–60%. Meanwhile, the proportion of females and patients with hypertension and type 2 diabetes was higher in the CMVD group than in the non-CMVD group (*P* < 0.05,Table 1).

**Table 1:** Comparison of general clinical data between CMVD group and non-CMVD group

Variable	CMVD group n = 175	Non-CMVD group n = 175	P value
Age,years	57.83 ± 10.94	57.68 ± 11.73	0.846
Female	110(62.86)	80(45.71)	0.029
Body Mass Index(kg/m^2^)	24.46 ± 12.76	24.11 ± 13.08	0.733
Heart rate at admission(bpm)	72.26 ± 11.28	71.65 ± 12.52	0.423
Hypertension	76(43.43)	39(22.28)	0.021
Type 2 Diabetes	95(54.29)	30(17.14)	0.017
Left ventricular ejection fraction between 35% and 60%	78(44.57)	52(29.71)	0.158
Smoke	80(45.71)	60(34.29)	0.379
Drinking	36(20.57)	26(14.86)	0.768

### Comparison of laboratory data between the two groups

Compared with the non-CMVD group, the CMVD group had higher levels of platelet count, C-reactive protein (Crp), and TG and ratio of TG/HDL-C but lower levels of albumin and HDL-C (*P* < 0.05, Table 2).

**Table 2:** Comparison of laboratory data between the CMVD group and the Non-CMVD group

Variable	CMVD group n = 175	Non-CMVD group n = 175	P value
White blood cells(10^9^/L)	5.96 ± 1.55	5.75 ± 1.62	0.249
Red blood cell(10^9^/L)	4.42 ± 0.43	4.35 ± 0.49	0.097
Hemoglobin(g/L)	134.86 ± 12.82	132.54 ± 13.77	0.133
Platelet count(10^9^/L)	201.33 ± 57.08	121.96 ± 57.78	0.043
Neutrophil(10^9^/L)	3.38 ± 1.46	3.25 ± 1.10	0.334
Creatinine(umol/l)	66.78 ± 14.62	64.24 ± 14.51	0.164
Crp(mg/L)	3.62 ± 6.58	2.10 ± 4.77	0.022
Albumin(g/L)	39.07 ± 3.17	43.15 ± 4.1	0.039
ALT(U/L)	18.64 ± 8.77	20.75 ± 8.09	0.085
AST(U/L)	23.07 ± 10.41	22.12 ± 11.93	0.194
HDL-C(mmol/l)	0.91 ± 0.22	1.28 ± 0.57	0.013
LDL-C(mmol/l)	2.39 ± 0.77	2.35 ± 0.79	0.563
TC(mmol/l)	4.43 ± 1.02	4.12 ± 0.97	0.144
TG[M(P_25_,P_75_),umol/L)]	2.37(1.81,3.29)	1.04(0.82,1.38)	0.006
FBG(mmol/l)	5.06 ± 1.81	4.77 ± 1.16	0.327
TG/HDL-C[M(P_25_,P_75_),umol/L)]	2.48(1.76,3.66)	0.92(0.61,1.24)	<0.001

ALT:Alanine aminotransferas; AST:Aspartate aminotransferase; Crp:C-reactive protein; FBG: Fasting blood glucose; HDL-C:High-density lipoprotein cholesterol; LDL-C:Low density lipoprotein cholesterol; TC:total cholesterol; TG:triglyceride

### Multivariate Logistic regression analysis

The logistic regression analysis was performed by involving clinical indicators with statistically significant differences, which showed that Crp, albumin, TG/HDL-C ratio, and sex were independent risk factors for CMVD (*P* < 0.05,Table 3).

**Table 3:** Binary Logistic regression analysis of risk factors for CMVD

Variable	B	SE	Wald	OR Value	95%Cl	P value
Hypertension	0.140	0.084	0.540	1.158	0.677–1.854	0.360
Diabetes	0.890	0.202	2.836	0.873	0.464–1.083	0.093
Blood platelet	0.428	0.251	1.294	1.273	0.925–1.861	0.255
TG	0.347	0.133	4.036	1.382	1.104–1.737	0.048
Crp	1.014	0.277	3.792	1.683	1.140–3.456	0.011
Albumin	0.722	0.330	4.824	2.052	1.080–3.905	0.028
HDL-C	0.295	0.154	4.019	0.730	0.447–0.979	0.033
TG/HDL-C	0.549	0.108	5.523	1.826	1.320–2.973	0.005
Sex	0.434	0.220	3.844	1.545	1.001–2.382	0.049

Crp:C-reactive protein; HDL-C:High-density lipoprotein cholesterol; TG: triglyceride

### The results of the ROC curve

**Fig. 2:**
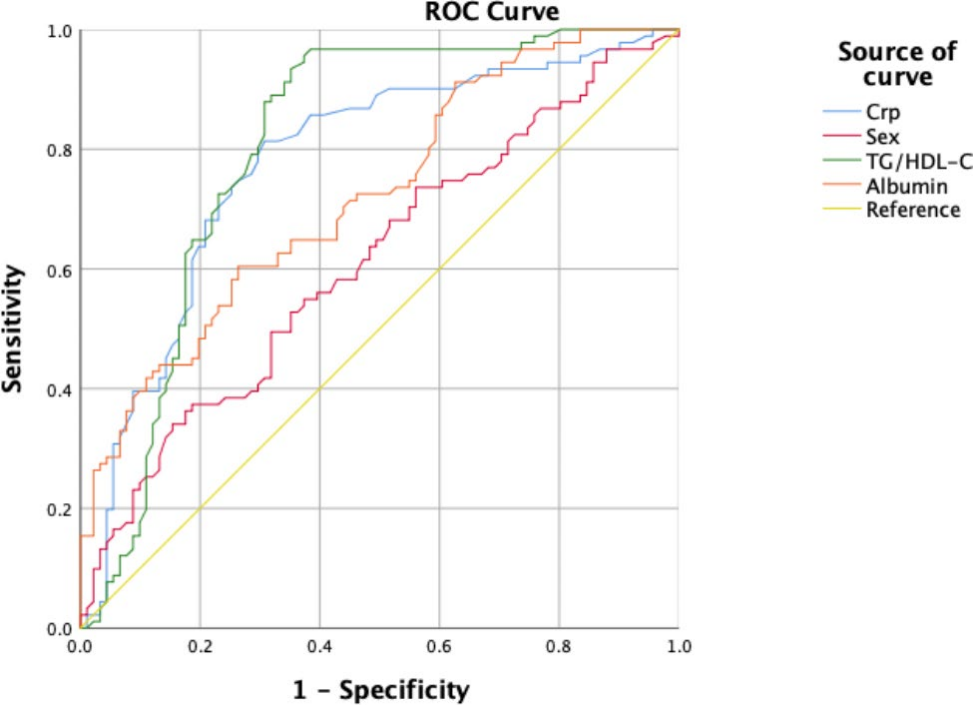
ROC Curve Analysis of HG/HDL-C,Sex,Crp and Albumin

The TG/HDL-C ratio had an area under ROC curve (AUC) value of 0.789 and 95% confidence interval (95%CI) of 0.718–0.859 (*P* < 0.001) higher than those of sex (0.651, 0.571–0.730, *P* < 0.001), albumin (0.722, 0.649–0.794, *P* < 0.001), and Crp (0.754, 0.681–0.827, *P* < 0.001). The results illustrated that the TG/HDL-C ratio had superior test efficiency. (Fig. 2)

## Discussion

CMVD represents a syndrome exhibiting objective evidence of exertional angina pectoris or myocardial ischemia attributed to structural abnormalities or dysfunction of anterior coronary arterioles and arterioles in the presence of various risk factors [12]. The abnormal structure and dysfunction of coronary microcirculation can affect cardiovascular metabolism, which exerts a substantial impact on the occurrence, development, evolution, and prognosis of cardiovascular disease. In turn, cardiovascular disease also causes and aggravates the structural abnormalities and dysfunction of coronary microcirculation [13]. At present, the clinical treatment of myocardial reperfusion usually involves coronary intervention and coronary artery bypass grafting. Nonetheless, These two treatments cannot solve the issue of abnormal myocardial blood perfusion caused by coronary microcirculation although they can open the narrowed epicardial coronary arteries in time. Accordingly, a simple index to identify coronary microcirculatory disease is useful for the early clinical intervention of CMVD, which has important clinical implications for improving the prognosis of patients.

CMVD-related factors have been extensively analyzed. For instance, the research by Li et al. [14] manifested markedly higher Crp levels in the CMVD group than in the non-CMVD group, suggesting the potential involvement of inflammation in the pathogenesis of CMVD. Gokce et al. [15] also observed that the platelet aggregation rate in the CMVD group was substantially higher than that in the non-CMVD group, indicating that platelet dysfunction may play a crucial role in CMVD pathogenesis. Another study [16] unveiled increased blood uric acid levels as one of the risk factors for CMVD patients. In addition, CMVD has also been revealed to correlate to obesity [17], smoking [18], hyperlipidemia, hypoalbuminemia, and insulin resistance [19]. Unfortunately, little is known about the risk factors related to CMVD. Meanwhile, no final conclusions have been reached regarding the indicators for identifying CMVD.

In our study, the CMVD group had a higher proportion of females and patients with hypertension and type 2 diabetes than the non-CMVD group, consistent with the results obtained by Bairey et al. [20]. Furthermore, we also noted that the level of platelet count, CRP, and TG was higher but the level of albumin and HDL-C was lower in the CMVD group than in the non-CMVD group. Similarly, a previous study [21] elucidated that the elevated platelet aggregation rate resulted in high shear stress, slowed blood flow, and accelerated endothelial cell damage, thus predisposing to microvascular dysfunction.

The increased expression of inflammatory factors, such as CRP, is associated with the aggravation of vascular endothelial oxidative stress and the decline in endothelial-mediated vascular function and the fibrinolytic capacity of the body, which are pivotal pathophysiological mechanisms underlying coronary microvascular lesions [22].

High TG levels and low HDL-C levels are implicated in the promotion of lipid peroxidation and endothelial cell damage, finally inducing microcirculation disturbance [23]. Moreover, low albumin levels are associated with systemic inflammation and excessive oxidative stress, which leads to microvascular diseases [24].

A prior study confirmed TG and HDL-C as the major indexes reflecting the blood lipid in the body, illustrating their implications in the occurrence of atherosclerosis [25]. The TG/HDL-C ratio is a novel composite index that can not only reflect the disorder of blood lipid metabolism, but also is related to insulin resistance [26]. As reported, the TG/HDL-C ratio has higher potentials than TG and HDL-C in reflecting the comprehensive level of lipid metabolism in the body and accurately predicting the risk of cardiovascular disease. Furthermore, the TG/HDL-C ratio has been validated as an independent risk factor for coronary heart disease, which is closely associated with the major adverse cardiovascular events in patients with coronary heart disease, including coronary heart disease death, acute myocardial infarction, and vascular remodeling following unstable angina pectoris [27].

A previous study in patients with inflammatory bowel disease unraveled that in contrast to healthy controls with CFR ≥ 2.5, patients with CFR < 2.5 had higher TG concentrations and lower HDL-C concentrations, indicating the potential association of TG and HDL-C with CMVD [28]. Accordingly, we speculated that the TG/HDL-C ratio is related to the pathogenesis of coronary microcirculatory disease.

Our study demonstrated that the TG/HDL-C ratio in the CMVD group was higher than that in the non-CMVD group. Our multivariate logistic regression analysis also indicated Crp,sex, albumin, and the TG/HDL-C ratio as independent risk factors for CMVD. Compared with that of Crp, sex, and albumin, the AUC value of the TG/HDL-C ratio was higher, indicating that the TG/HDL-C ratio might be more efficient in identifying CMVD.

The mechanism by which TG/HDL-C is associated with CMVD has been barely probed, which may be related to insulin resistance. Insulin resistance has been reported to directly exacerbate vascular endothelial cell injury, activate the damage/repair mechanism of the body, and promote the aggregation of platelets, white blood cells, and other inflammatory cells [29]. Meanwhile, the number of vascular smooth muscle cells increases compensatorily and the content of vasodilator factors in the body decreases in response to insulin resistance, thus contributing to the imbalance of vascular contraction and expansion, vascular endothelial damage, and microvascular endothelial dysfunction [30]. Another study revealed that the expression of serum adiponectin and adhesion molecules was also enhanced in vivo in the presence of insulin resistance, which altered vascular regulation in the body and accelerated vascular endothelial injury and myocardial ischemia [31].

## Conclusion

In conclusion, the TG/HDL-C ratio can be utilized as an independent risk factor for CMVD occurrence, thereby providing certain clinical reference data for the evaluation of coronary microcirculation function in patients with non-large vascular obstructive angina pectoris. Meanwhile, the composite index TG/HDL-C composed of their ratio has greater diagnostic value in predicting CMVD occurrence than single blood lipid indexes, which, thereby, is clinically valuable.

### Limitations

There are still some limitations in this study. First, our research is a single-center study with a small sample size. Therefore, selection bias cannot be ruled out. Second, the specific mechanism of TG/HDL-C on coronary microcirculatory disease remains poorly identified. Accordingly, further experimental verification is warranted to obtain more supporting evidence and clarify the related mechanism. Third, the follow-up of patients participating in this study is still in progress. Consequently, no further analysis of their prognosis has not been conducted. In the future, prospective multi-center studies with larger sample sizes are warranted for further validating our conclusions.

## Data Availability

The datasets used during the present study are available from the corresponding author upon reasonable request.

## Abbreviations

ATP: Adenosine triphosphate
CAG: Coronary angiography
CMVD: Coronary microvascular disease
LCS: Coronary sinus
LAR: Aortic root
ISDN: Intracoro- nary isosorbide mononitrate
ROC: Receiver operating characteristic
